# Low-dose ultrahigh-resolution PCCT enhances subsolid nodule characterization

**DOI:** 10.1007/s11547-025-02057-0

**Published:** 2025-07-29

**Authors:** Qinqin Yan, Fuhua Yan, Qi Lin, Qiqi Cao, Yajie Zhang, Xiaoyan Chen, Bernhard Schmidt, Zhihan Xu, Wenjie Yang

**Affiliations:** 1 Department of Radiology, Ruijin Hospital, Shanghai Jiaotong University School of Medicine, No.197, Ruijin Second Road, Huangpu District, Shanghai, China; 2 Department of Thoracic Surgery, Ruijin Hospital, Shanghai Jiaotong University School of Medicine, No.197, Ruijin Second Road, Huangpu District, Shanghai, China; 3 Department of Pathology, Ruijin Hospital, Shanghai Jiaotong University School of Medicine, No.197, Ruijin Second Road, Huangpu District, Shanghai, China; 4 Siemens Healthcare GmbH, Computed Tomography, Forchheim, Germany; 5 Department of CT Collaboration, Siemens Healthineers Ltd, Shanghai, China

**Keywords:** Subsolid nodules, Lung adenocarcinoma, Ultrahigh resolution, Photon-counting CT

## Abstract

**Purpose:**

To characterize invasion-associated CT features in pulmonary subsolid nodules using low-dose ultrahigh-resolution (UHR) photon-counting CT (PCCT) images and evaluate UHR’s diagnostic superiority over standard high-resolution (HR) images.

**Methods:**

Patients with subsolid lung adenocarcinoma were recruited for chest scan on PCCT to obtain UHR and standard HR images between November 2023 and May 2024. Nodule characteristics were visually assessed and histogram features were extracted from each nodule. Image quality and radiation dose at previous energy-integrating detector CT (EID-CT) of 30 patients were compared with those of PCCT. Differences between UHR and standard HR, PCCT and EID-CT were compared using paired McNemar-test or paired Wilcox-test.

**Results:**

One hundred and eighty-four patients with 203 subsolid nodules were collected including 77 precursors, 77 minimally invasive adenocarcinoma (MIA) and 49 IA. UHR significantly outperformed standard HR in revealing CT findings including larger nodular diameter and solid-component diameter, more frequency of heterogeneous attenuation, lobulation, bubble-like sign, air bronchogram, pleural indentation and vascular sign (all *P* < 0.05). Additionally, UHR images exhibited significantly greater value in histogram-derived parameters compared to standard HR images (all *P* < 0.05), except for “Median,” “Minimum.” Furthermore, the radiation dose in PCCT was half of that in EID-CT (effective dose: 1.32 ± 0.27 vs. 3.85 ± 1.65/mSv, *P* < 0.001. CDTI_vol_: 2.97 ± 0.53 vs. 6.90 ± 2.97/mGy, *P* < 0.001), with image quality significantly better in PCCT.

**Conclusion:**

The UHR protocol on PCCT provides a magnified perspective to reveal CT characteristics of invasive growth in subsolid LUAD, previously undetectable on standard HR images, achieving halved radiation dose and better image quality than EID-CT.

**Supplementary Information:**

The online version contains supplementary material available at 10.1007/s11547-025-02057-0.

## Introduction

The most common histological type of non-small cell lung cancer is lung adenocarcinoma (LUAD), with an increasing incidence due to low-dose CT screening. Usually, early stage LUAD appears as subsolid nodules on CT images. However, such nodules histopathologically exhibit varying levels of biological aggressiveness, including invasive lesions such as invasive adenocarcinoma (IA) and minimally invasive adenocarcinoma (MIA), and their precursors such as adenocarcinoma in situ (AIS) and atypical adenomatous hyperplasia (AAH) [1, 2]. Thus, the presurgical radiological categorization of subsolid nodules is critical for treatment planning. The precursors are not recommended for surgical resection, whereas MIA and IA usually require sublobectomy or lobectomy resection, respectively [3–5].

Subsolid nodules should be evaluated following pulmonary guidelines that consider both the nodule size and the solid component size. Generally, an increased maximum diameter of both the solid component and nodule indicates a higher probability of malignancy and requires a cautious follow-up [6, 7]. However, approximately 8–23% of IA remains even in subcentimeter ground-glass nodules (GGNs) (with a maximum diameter less than 10 mm) [8–10]. Elevated radiographic attenuation values [11], and presence of CT morphological characteristics, such as irregular outline, spiculation, and bubble-like appearance, demonstrate diagnostic utility in differentiating subsolid pulmonary nodules [9, 12, 13]. Nevertheless, accurately identifying invasive lesions using the common high-resolution energy-integrating detector CT (EID-CT), even for experienced radiologists, still remains difficult.

Recently, the technical evolution of detectors using semiconductor materials (e.g., cadmium telluride) has allowed the development of new photon-counting detectors (PC detectors), enabling photon-counting CT (PCCT) systems that directly convert absorbed X-rays to electronic signals without reflective septa [14, 15]. Therefore, PCCT has higher geometric dose efficacy and eliminates electric noise. Compared with a 278-µm in-plane limiting resolution in EID-CT [16], the latest commercial dual-source PCCT scanner (NAEOTOM Alpha, Siemens Healthineers) can provide a 125-µm in-plane spatial resolution and 0.34-mm longitudinal resolution with 120 $\times$ 0.2-mm detector collimation [17]. Besides, it can obtain the ultrahigh-resolution (UHR) images at the prerequisite of low dose. Studies have shown that UHR imaging produced by PCCT allows better visualization of almost all lung structures and facilitates the evaluation of interstitial lung diseases [18, 19]. However, whether UHR images from PCCT can improve the accurate categorization of pulmonary subsolid nodules remains unknown.

This study systematically evaluates the superiority of PCCT-derived UHR imaging in characterizing subsolid LUAD, employing a comparative analysis against standard high-resolution (HR) images.

## Materials and methods

### Participants

**Fig. 1:**
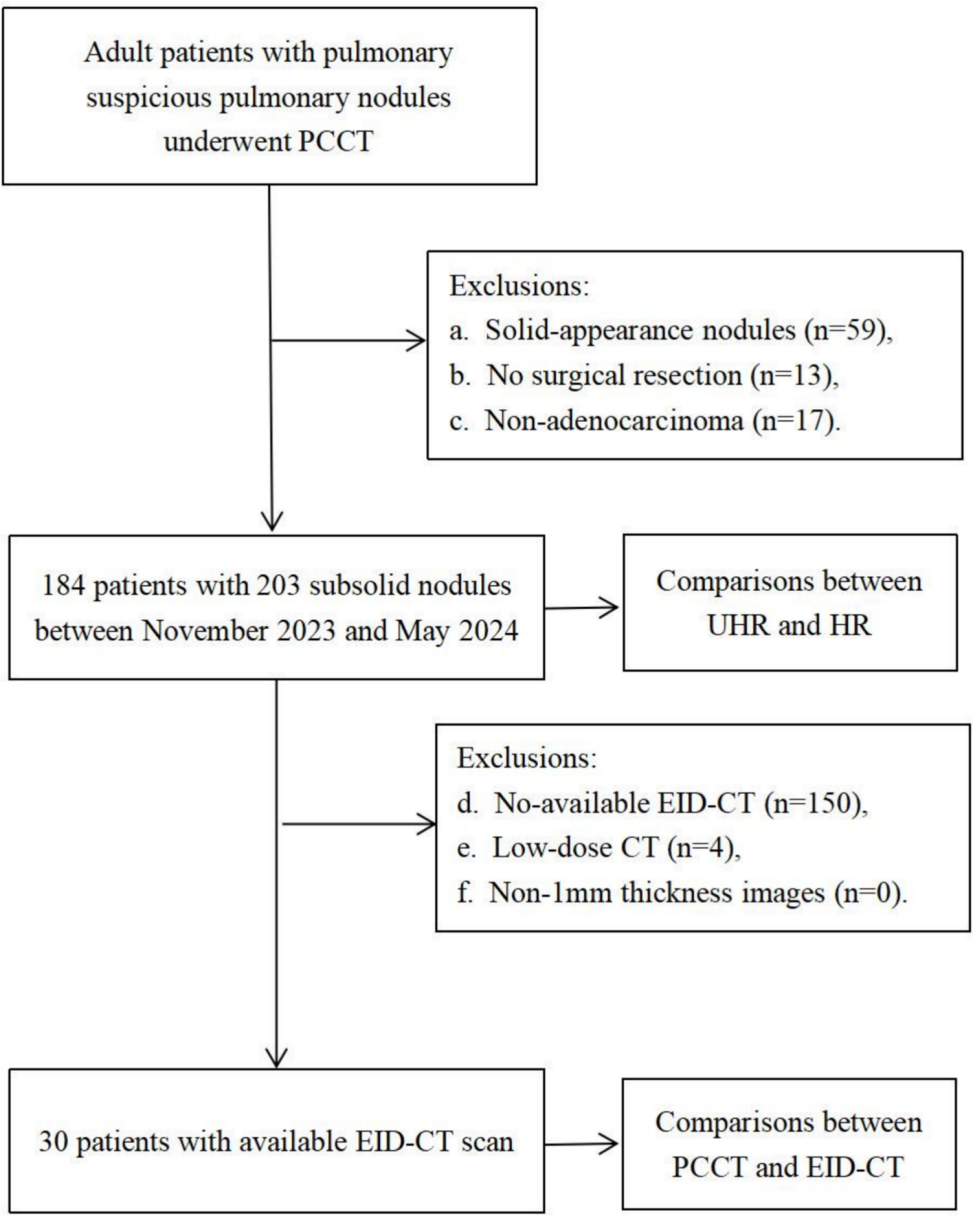
The workflow of patients’ enrollment

This retrospective study was approved by the ethics institutional review board (approval number: AF0406/14.0/2023–03-01), and informed consent was waived. From November 2023 to May 2024, consecutive adult participants (≥ 18 years old) with suspicious pulmonary nodules who required routinely presurgical PCCT evaluation were enrolled. The exclusion criteria were as follows: (a) appearing as solid lesions, (b) no surgical resection within 3 months, (c) and a non-adenocarcinoma. Moreover, to facilitate a comparative evaluation of image quality and radiation dose, images from participants who had previously undergone chest plain CT scans at our institution were gathered (Fig. 1).

### Histopathology

All histopathological specimens were stained using hematoxylin and eosin (H&E). Two pathologists with over 20 years of experience observed these specimens and performed pathological classifications according to the International Association for the Study of Lung Cancer [1, 2]. Those objections were determined via discussions.

### CT protocol

A first-generation dual-source PCCT scanner (NAEOTOM Alpha, version Syngo CT VA50 SP1; Siemens Healthineers) was adopted with scanning parameters as follows: ultrahigh-resolution mode with collimation 120 $\times$ 0.2 mm, 140 kVp with tin filtration, pitch factor of 3.2, rotation time of 0.25 s, and automated tube current modulation (CAREDose 4D) with image quality level 54 for noncontrast imaging. The images were reconstructed including 1.0-mm thickness / 1.0-mm increment with a 512 $\times$ 512 matrix (standard HR), and 0.2-mm thickness / 0.2-mm increment with a 1024 $\times$ 1024 matrix (UHR). The field of view was range from 30 to 45 cm. A lung convolution kernel (Bl60) and a quantum iterative reconstruction algorithm at the level of 4 were used for both image series. The thoracic plain scans on EID-CT were performed with 80–120 kVp, auto-modulation tube current, a 512 $\times$ 512 matrix, and 1.0-mm thickness (Table S1).

### CT findings interpretation

All images were redacted series information and then randomly assigned to two thoracic radiologists with over six years of experiences. They independently assessed the CT findings and image quality using Siemens Syngo.Via workstation (version VB40). Standard window presets were applied with lung window settings (window level [(WL] = -600 HU, window width [WW] = 1200 HU) and mediastinal settings (WL = 40 HU, WW = 350 HU). Then one senior radiologist with 22 years of experiences resolved any discrepancies. All radiologists were blind to the histopathology and image information.

Measurements of the attenuation value (CT value), standard deviation (SD), maximum nodule diameter, and maximum solid-component diameter were conducted on axial CT images, with resultant averages. A pure GGN (pGGN) is the nodule with hazy density without obscuring the underlying pulmonary vasculature. In contrast, a mixed GGN (mGGN) consists of both ground-glass attenuation and solid component. Lobulation indicates an irregular outline of the nodule. The spiculation is a radical opacity arising from the nodular surface. The air bronchogram sign depicts the bronchus traversing the nodule. Pleural indentation exhibited one or multiple lines linking the nodular surface to the pleura, presenting as a bridge- or rabbit ear-like configuration. The vascular sign denotes aggregated or dilated vessels within the nodule whose diameters exceeds those of the proximal vessels.

### Histogram feature extraction

The histogram features were extracted using dedicated software tool (Radiomics V1_4_0 Prototype, Siemens Healthineers) running on a research platform (Syngo Via, Frontier, Siemens Healthineers; Version VB40). The volume of interest (VOI) was automatically segmented at the lung window (WL, -600 HU. WW, 1200 HU), then was reviewed and modified by the senior radiologist blinding to histopathology and image information.

### Image quality

The signal-to-noise ratio (SNR), contrast-to-noise ratio (CNR) and the noise were compared among standard HR on EID-CT, standard HR on PCCT, and UHR on PCCT.

**Fig. 2:**
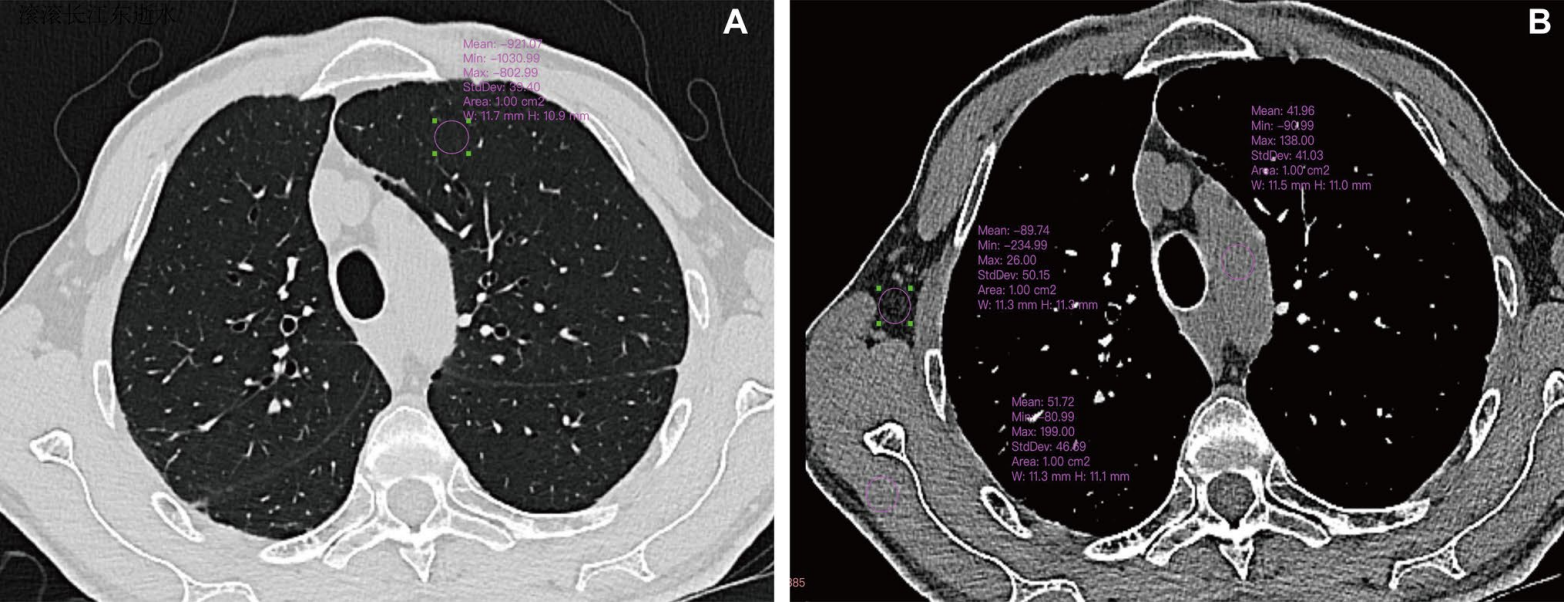
An example of image quality assessment. Signal-to-noise ratio (SNR) = ∣CT _lung_ = (-921HU) / SD _lung_ = (39HU) ∣ = 23.6, Contrast-to-noise ratio (CNR) = ∣ (CT _artery_ = (42HU) – CT _muscle_ = (52HU)) / SD _fat_ = (50HU) ∣ = 0.2

The circular region of interests (ROIs) with an area of 100 mm^2^ were placed on the left upper lobe (CT _lung_ ± SD _lung_) avoiding the vessel and bronchi at the lung window (WL, -600 HU. WW, 1200 HU), aortic arch (CT _artery_ ± SD _artery_), subscapularis muscle (CT _muscle_ ± SD _muscle_), and axillary fat (CT _fat_ ± SD _fat_) at the mediastinal window (WL, 40 HU. WW, 350 HU), respectively, to obtain an attenuation value and SD (as shown in Fig. 2). Those ROIs were copied to standard HR from EID-CT, standard HR from PCCT, and UHR from PCCT. The SNR and CNR were calculated as follows: SNR = ∣CT _lung_ / SD _lung_∣, CNR = ∣ (CT _artery_ – CT _muscle_) / SD _fat_∣.

### Radiation dose

Volume CT dose index (CTDI_vol_) and effective dose (ED) were calculated and compared between EID-CT and PCCT. The CTDI_vol_ represents the average radiation dose over the whole scanning volume, whereas the ED is calculated using the dose-length product (DLP) multiplied by 0.014.

## Statistical analysis

The R language software (version 4.3.1) was used to conduct the statistical analysis. Continuous variables were presented as medians with interquartile ranges (IQR) or means ± SD, and categorical variables were expressed as numbers with percentages, respectively. The Kruskal–Wallis test was used for comparisons among IA, MIA, and precursors groups. The paired Wilcoxon signed-rank test or McNemar test was implemented to evaluate the differences between EID-CT and PCCT, as well as between standard HR and UHR images for continuous and categorical variables, respectively. The inter-observer agreements on continuous and categorical variables were evaluated using intraclass correlation coefficient and kappa analysis, respectively. The results were expressed with percent 95 confidence interval (95%CI). Statistical significance was set at *P* < 0.05 (two sides).

## Results

### Patient characteristics

Of the 273 participants screened, 89 were excluded from the study including: appearing as solid lesions (n = 59), no surgical resection (n = 13), or a non-adenocarcinoma (n = 17). Thus, 184 participants (male, n = 54; female, n = 130; mean age, 54.0 years ± 13.7) with 203 subsolid nodules were included (Fig. 1); the nodules were pathologically confirmed as AAH (n = 4), AIS (n = 73), MIA (n = 77), and IA (n = 49), with the mean diameter of 11.2 ± 4.7 mm. Sex, smoking history, and nodular location had no statistical significance among the precursors, MIA, and IA groups (all *P* > 0.05). However, older age, larger diameter of both nodule and solid component, and radiographic type of mGGN were more frequently observed in advanced pathological grade (all *P* < 0.05) (Table 1).

**Table 1:** Characteristics of the subsolid nodules

	IA (n = 49)	MIA (n = 77)	AAH-AIS (n = 77)	*P*-value
Sex, M (%)	15 (30.6)	22 (28.6)	17 (22.1)	0.507
Age, mean (SD) / y	60.6 (11.2)	53.8 (13.8)	48.7 (13.8)	< 0.001
Smoking history / yes, (%)	2 (4.1)	5 (6.5)	3 (3.9)	0.723
Lung lobe (%) LLL LUL RLL RML RUL	2 (4.1) 21 (42.9) 8 (16.3) 5 (10.2) 13 (26.5)	11 (14.2) 20 (26.0) 12 (15.6) 4 (5.2) 30 (39.0)	10 (13.0) 19 (24.7) 12 (15.6) 4 (5.2) 32(41.5)	0.656
Nodular diameter, mean (SD) / mm	17.1 (4.9)	10.7 (3.1)	8.5 (2.4)	< 0.001
Nodular diameter subgroup (%) 5-10 mm 10-15 mm 15-30 mm	5 (10.2) 11 (22.4) 33 (67.3)	43 (55.8) 27 (35.1) 7 (9.1)	64 (83.1) 11 (14.3) 2 (2.6)	< 0.001
Solid-component diameter, mean (SD) / mm	8.3 (5.6)	2.9 (3.0)	0.6 (1.2)	< 0.001
Radiographic type (%)* pGGN mGGN	6 (12.2) 43 (87.8)	30 (39.0) 47 (61.0)	62 (80.5) 15 (19.5)	< 0.001

IA, invasive adenocarcinoma. MIA, minimally invasive adenocarcinoma, AIS, adenocarcinoma in situ. AAH, atypical adenocarcinoma hyperplasia. SD, standard deviation. LLL, left lower lobe. LUL, left upper lobe. RLL, right lower lobe. RML, right middle lobe. RUL, right upper lobe. pGGN, pure ground-glass nodule. mGGN, mixed ground-glass nodule

^*^ The radiographic type of subsolid nodules were assessed on standard HR images

### Comparisons of image quality and radiation dose

The EID-CT chest images of 30 participants were retrieved for image quality and dose comparisons. The average time interval between the two scans was 4.3 months.

The SNR of both UHR (15.4 ± 2.5) and standard HR (19.7 ± 3.6) on PCCT was significantly higher than that of EID-CT (14.7 ± 5.9) (*P* < 0.001, *P* = 0.026). Furthermore, the noise of both UHR (61.1 ± 9.1) and standard HR (47.9 ± 7.6) on PCCT were significantly lower than that of EID-CT (67.6 ± 20.5) (*P* = 0.045, *P* < 0.001). In terms of CNR, UHR on PCCT (0.25 ± 0.10) were comparable to that of EID-CT (0.25 ± 0.15) (*P* = 0.863), although both were inferior to standard HR on PCCT (0.34 ± 0.12) (*P* < 0.001, *P* = 0.007).

The ED of PCCT was significantly lower than that of EID-CT (average: 1.32 ± 0.27 vs. 3.85 ± 1.65 / mSv, IQR: 1.23 (1.09, 1.47) vs. 3.67 (2.87, 4.36) / mSv, *P* < 0.001). Meanwhile, the CTDI_vol_ of PCCT was also evidently lower than that of EID-CT (average: 2.97 ± 0.53 vs. 6.90 ± 2.97 / mGy, IQR: 2.8 (2.5, 3.3) vs. 7.3 (4.2, 9.0) / mGy, *P* < 0.001).

### Comparisons of CT findings between UHR and standard HR on PCCT

**Fig. 3:**
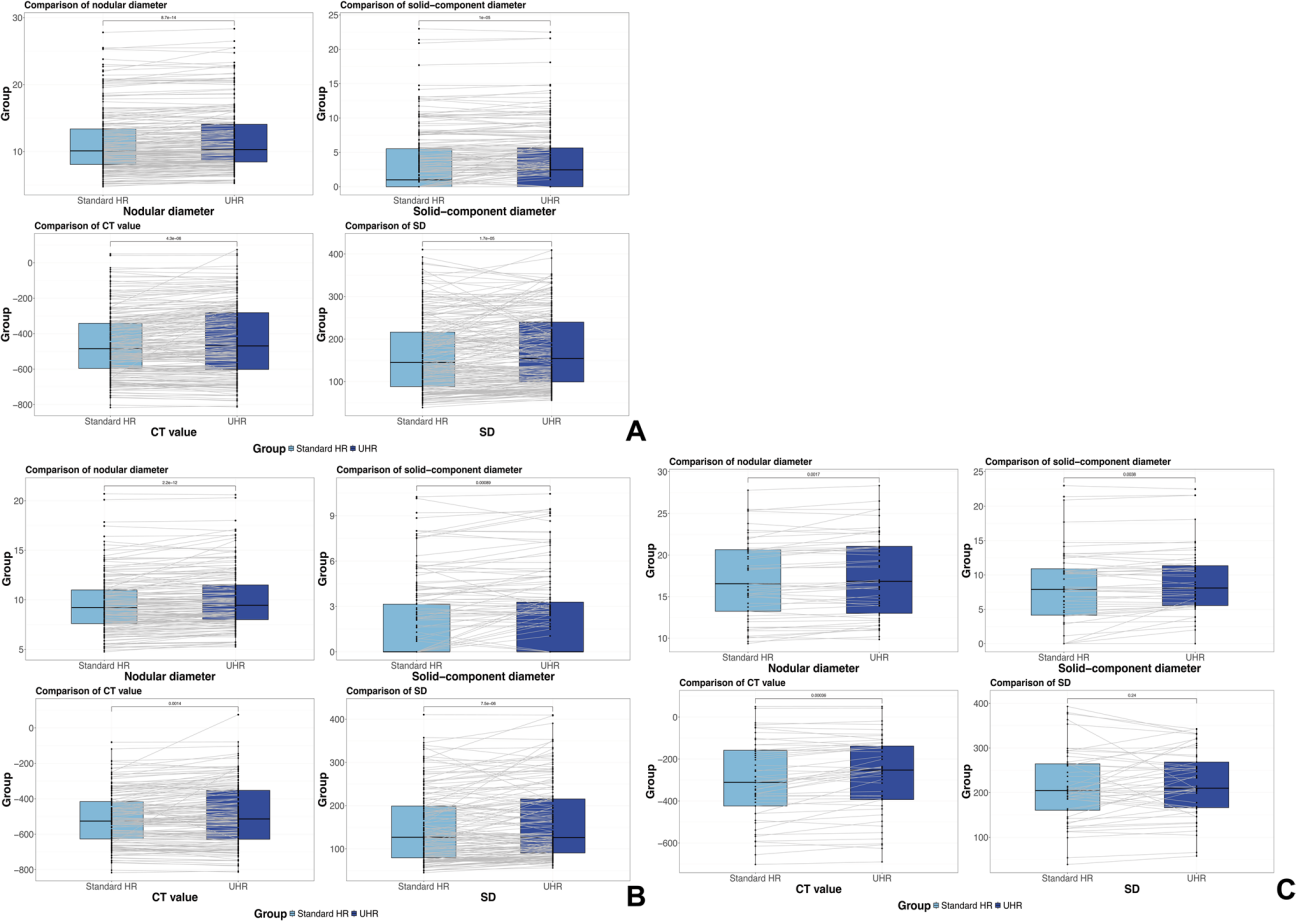
Paired boxplots comparing nodular diameter, solid-component diameter, CT value, and its standard deviation (SD) between ultrahigh-resolution (UHR) and standard HR images across all LUAD subtypes (**A**), in the pre-IA subtype (**B**) and in the IA subtype (**C**)

**Fig. 4:**
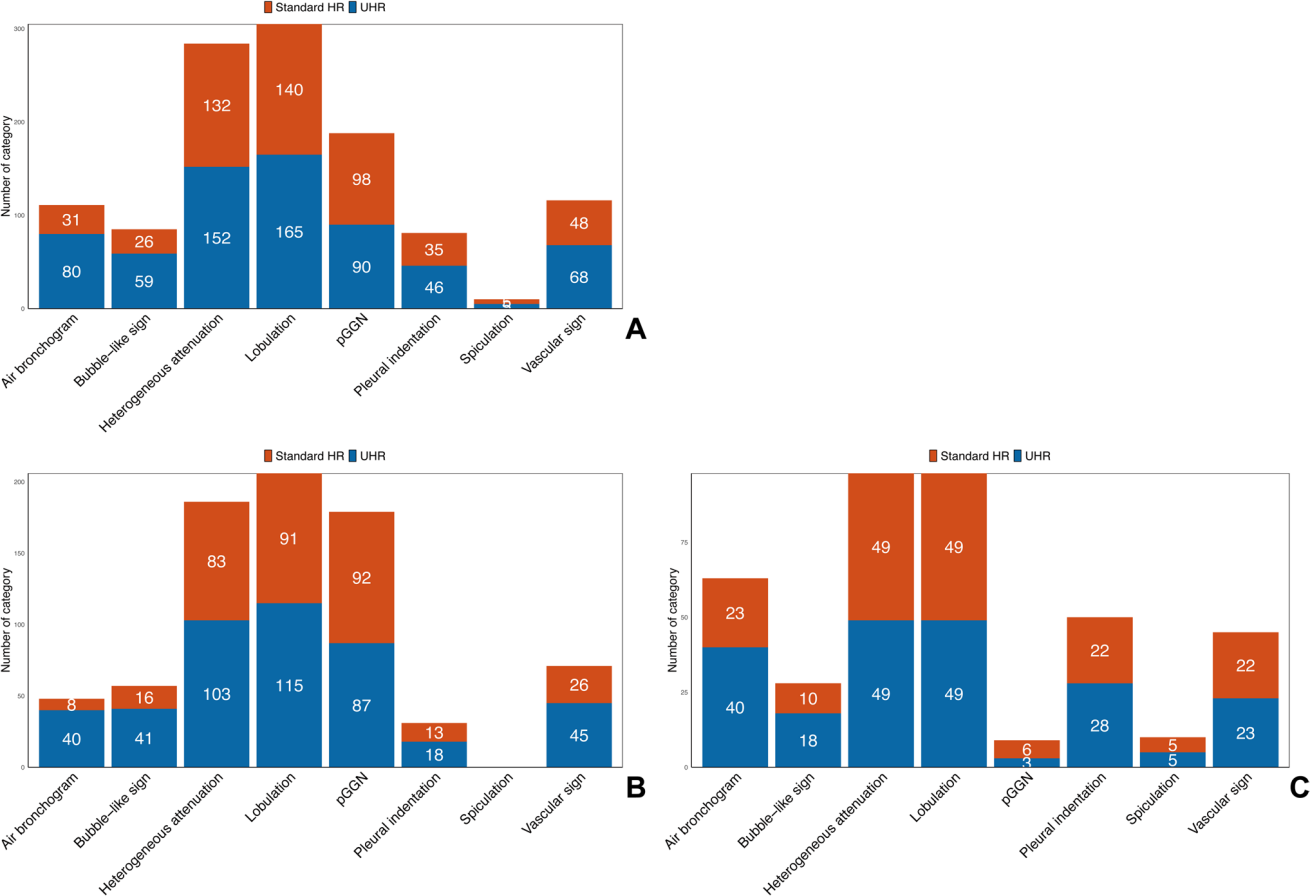
Bar charts comparing radiographic signs between UHR and standard HR images across all LUAD subtypes (**A**), in the pre-IA subtype (**B**) and in the IA subtype (**C**), respectively

**Fig. 5:**
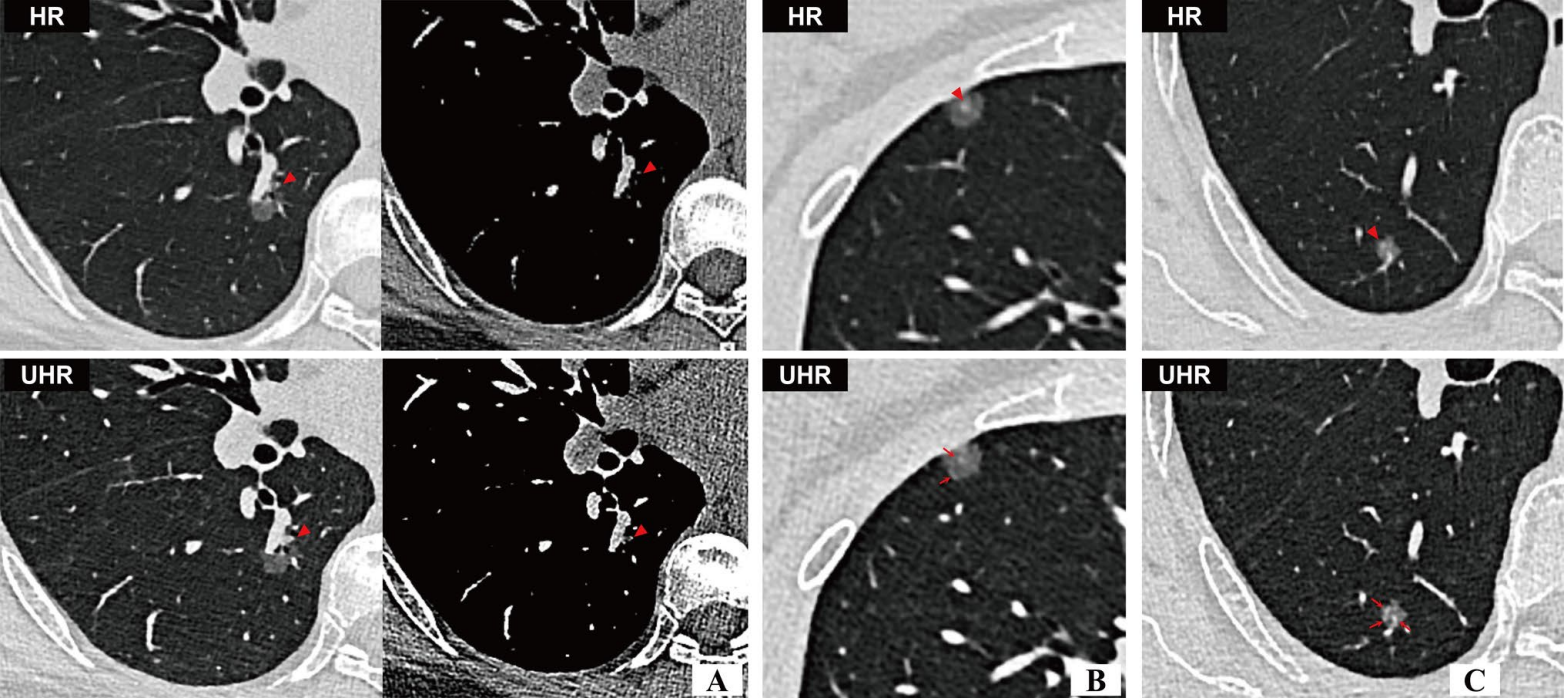
**A** An elderly female pathologically confirmed with IA in the right lower lobe. UHR images revealed a subtle solid component on both lung and mediastinal window settings (triangle), however, this critical sign was obscured on standard HR images, leading to misclassification of a pure ground-glass nodule (pGGN) and underestimation of the invasiveness of the LUAD. **B** A middle-aged woman with AIS in the right middle lobe. On standard HR images, the nodule exhibited a heterogeneous appearance with central subtle solid component (triangle). Contrarily, UHR images revealed this central component corresponds to a microvessel (arrow). An another microvessel was found at nodule periphery (arrow). **C** an elderly woman with AIS in the right lower lobe. On standard HR images, the peripheral solid microfoci (triangle) was initially classified as a mixed GGN category. However, UHR images revealed this structure corresponds to a microvascular component

The inter-observer consistency of visual assessment on UHR and standard HR images was presented in Table S2. On UHR images, solid components were identified in 11 nodules that initially presented as pGGNs on standard HR images which were pathologically confirmed to be three IA, six MIA and two AIS. Contrarily, three mGGNs on standard HR images were recategorized as pGGNs on UHR images including two AIS and one MIA. There was marginally difference in categorization of pGGN and mGGN (*P* = 0.061). As shown in Table 2, Figs. 3A and 4A, UHR images revealed significantly larger measurements of nodular diameter (*P* < 0.001), solid-component diameter (*P* < 0.001), CT attenuation value (*P* < 0.001) and its SD (*P* < 0.001), as well as higher frequencies of heterogeneous attenuation (*P* < 0.001), signs of lobulation (*P* < 0.001), bubble-like sign (*P* < 0.001), pleural indentation (*P* = 0.003) and vascular sign (*P* = 0.002) and air bronchogram (*P* < 0.001), compared to standard HR images. Figure 5 shows cases of radiographic types being misclassified.

**Table 2:** Comparison of CT findings between UHR and standard HR on PCCT

	UHR, % (n)	Standard HR, % (n)	Consistency	*P* value
Radiographic type, pGGN	44.3 (90)	48.3 (98)	0.861	0.061
Heterogeneous attenuation	74.9 (152)	65.0 (132)	0.672	< 0.001
Lobulation	81.3 (165)	69.0 (140)	0.565	< 0.001
Spiculation	2.5 (5)	2.5 (5)	1.000	NA
Bubble-like sign	29.1 (59)	12.8 (26)	0.509	< 0.001
Air bronchogram	39.4 (80)	15.3 (31)	0.392	< 0.001
Pleural indentation	22.7 (46)	17.2 (35)	0.830	0.003
Vascular sign	33.5 (68)	23.6 (48)	0.541	0.002
Nodular diameter /mm IQR Mean ± SD	10.3 (8.5, 14.1) 11.8 ± 4.8	10.1 (8.1, 13.4) 11.4 ± 4.8	0.983	< 0.001
Solid-component diameter /mm IQR Mean ± SD	2.5 (0, 5.7) 3.6 ± 4.6	1.0 (0, 5.6) 3.3 ± 4.5	0.295	< 0.001
CT value / HU IQR Mean ± SD	-469 (-602, -282) -441 ± 199	-485 (-596, -341) -460 ± 184	0.947	< 0.001
SD / HU IQR Mean ± SD	155 (99, 240) 174 ± 87	145 (89, 216) 163 ± 86	0.875	< 0.001

pGGN, pure ground-glass nodule. SD, standard deviation. HR, high resolution. UHR, ultrahigh resolution. IQR, interquartile range. HU, Hounsfield unit

For the pre-IA lesions (Table 3, Figs. 3B and 4B), more heterogeneous attenuation (*P* < 0.001), lobulation (*P* = 0.027), bubble-like sign (*P* < 0.001), air bronchogram (*P* < 0.001) and vascular sign (*P* = 0.002) were observed on UHR images, compared to standard HR images. However, no spiculation was discovered in pre-IA lesions on both UHR and HR images. Additionally, the pre-IA lesions on UHR images presented significantly larger nodular diameter (*P* < 0.001), solid-component diameter (*P* < 0.001), CT value (*P* = 0.001) and its SD (*P* < 0.001), compared to standard HR images.

**Table 3:** Comparisons of CT findings between UHR and standard HR across LUAD subtypes

	UHR, % (n)	Standard HR, % (n)	Consistency	*P* value
*Comparisons in the pre-IA subtype*				
Radiographic type, pGGN	56.5 (87)	59.7 (92)	0.853	0.228
Heterogeneous attenuation	66.9 (103)	53.9 (83)	0.620	< 0.001
Lobulation	75.3 (116)	59.1 (91)	0.514	< 0.001
Spiculation	0 (0)	0 (0)	1.000	NA
Bubble-like sign	26.6 (41)	10.4 (16)	0.462	< 0.001
Air bronchogram	26.0 (40)	5.2 (8)	0.210	< 0.001
Pleural indentation	11.7 (18)	8.4 (13)	0.821	0.074
Vascular sign	29.2 (45)	16.9 (26)	0.359	0.002
Nodular diameter /mm IQR Mean ± SD	9.4 (8.0, 11.5) 10.0 ± 3.0	9.2 (7.6, 11.0) 9.6 ± 3.0	0.959	< 0.001
Solid-component diameter /mm IQR Mean ± SD	0 (0, 3.3) 1.9 ± 2.7	0 (0, 3.2) 1.7 ± 2.6	0.953	< 0.001
CT value / HU IQR Mean ± SD	−514 (−628, −352) −494 ± 173	−526 (−626, −416) −510 ± 151	0.923	0.001
SD / HU IQR Mean ± SD	126 (90, 215) 159 ± 86	127 (79, 199) 147 ± 81	0.898	< 0.001
*Comparisons in the IA subtype*				
Radiographic type, pGGN	6.1 (3)	12.2 (6)	0.633	0.248
Heterogeneous attenuation	100 (49)	100 (49)	1.000	NA
Lobulation	100 (49)	100 (49)	1.000	NA
Spiculation	10.2 (5)	10.2 (5)	1.000	NA
Bubble-like sign	36.7 (18)	20.4 (10)	0.600	0.013
Air bronchogram	81.6 (40)	46.9 (23)	0.244	< 0.001
Pleural indentation	57.1 (28)	44.9 (22)	0.755	0.041
Vascular sign	46.9 (23)	44.9 (22)	0.877	1.000
Nodular diameter /mm IQR Mean ± SD	16.9 (13.0, 21.1) 17.5 ± 5.0	16.6 (13.3, 20.7) 17.1 ± 4.9	0.980	0.002
Solid-component diameter /mm IQR Mean ± SD	8.1 (5.6, 11.4) 8.9 ± 5.2	7.9 (4.2, 10.9) 8.3 ± 5.6	0.965	0.004
CT value / HU IQR Mean ± SD	−252 (−393, −138) −275 ± 185	−311 (−424, −158) −304 ± 191	0.955	< 0.001
SD / HU IQR Mean ± SD	210 (167, 269) 220 ± 74	204 (160, 264) 214 ± 83	0.800	0.244

pGGN, pure ground-glass nodule. SD, standard deviation. HR, high resolution. UHR, ultrahigh resolution. IQR, interquartile range. HU, Hounsfield unit

**Fig. 6:**
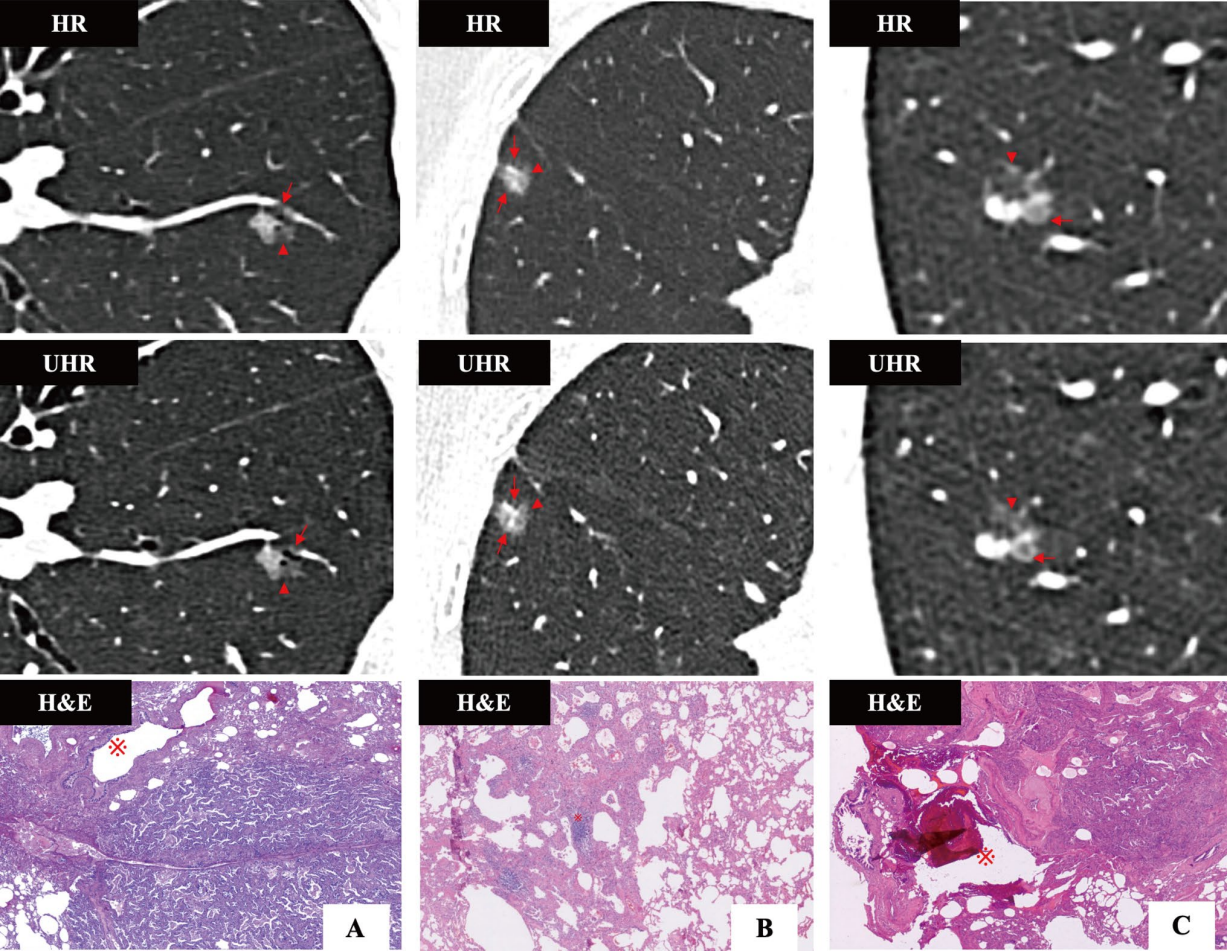
**A** An elderly male with MIA. On UHR images, the air bronchogram (arrow) is clearly shown within the nodule. In contrast, standard HR images only demonstrate an ill-defined hypoattenuating area adjacent to the nodule. Besides, UHR images enable more precise delineation of the nodular margin (arrowhead) compared to standard HR images. **B** A middle-aged female with MIA. UHR images more clearly demonstrate lobulation (arrowhead), heterogeneous attenuation, a coarse tumor–lung interface, and microvessels compared to standard HR image. Corresponding H&E image ($\times$ 200 times) shows tumor cell nests and widen lobular septa (※). **C** An elderly female with IA. Compared to standard HR, UHR images demonstrate enhanced visualization of the bubble-like sign (arrow), nodular margin (arrowhead), and air bronchogram. The bubble-like appearance corresponds to the air-containing area (※) in H&E image ($\times$ 200 times)

As for IA (Table 3, Figs. 3C and 4C), fewer pGGNs were observed on UHR images than on standard HR images (*ICC* = 0.555), although there was no statistical difference (6.1% vs. 14.3%, *P* = 0.248). Meanwhile, no significant differences were observed between UHR and standard HR images in revealing spiculation, lobulation, heterogeneous attenuation and vascular sign (all *P* > 0.05). However, UHR images demonstrated significantly higher detection rates of bubble-like sign (*P* = 0.013), air bronchogram (*P* < 0.001) and pleural indentation (*P* = 0.041) (Fig. 6). In addition, UHR images exhibited larger measurements in nodular diameter (*P* = 0.002), solid-component diameter (*P* = 0.004) and CT value (*P* < 0.001) compared to standard HR images. No significant difference was observed in SD between UHR and standard HR images (*P* = 0.244).

### Comparisons of histogram features between UHR and standard HR images

As presented in Table 4, UHR and standard HR images showed no differences in “Median” and “Minimum” across all LUAD subtypes (both *P* > 0.05); however, UHR images exhibited significantly larger values in histogram features reflecting signal intensity, heterogeneity and energy, alongside reduced “10Percentile” and “Uniformity” (all *P* < 0.05). Similar observations occurred in both IA and pre-IA subtypes (Table 4), with exceptions of “Kurtosis” and “Skewness” showing no differences in the pre-IA subtype and “Minimum” demonstrating statistical significance in both IA and pre-IA subtypes.

**Table 4:** Comparisons of histogram features between UHR and standard HR

	UHR	Standard HR	Consistency	*P* value
*Comparisons across all LUAD subtypes*				
10Percentile	−810 ± 89	−775 ± 92	0.831	< 0.001
90Percentile	−228 ± 224	−278 ± 232	0.937	< 0.001
Energy	11,209,159,060 ± 17,870,037,714	269,270,070 ± 447,693,808	−0.115	< 0.001
Entropy	5.0 ± 0.5	4.7 ± 0.5	0.750	< 0.001
Interquartile Range	280 ± 131	240 ± 117	0.909	< 0.001
Kurtosis	7.0 ± 4.9	6.5 ± 4.9	0.861	0.011
Maximum	785 ± 399	338 ± 363	0.330	< 0.001
Mean	−548 ± 133	−555 ± 131	0.974	< 0.001
Mean Absolute Deviation	186 ± 66	160 ± 63	0.881	< 0.001
Median	−590 ± 143	−593 ± 139	0.977	0.766
Minimum	−1022 ± 12	−1012 ± 116	0.482	0.389
Range	1807 ± 401	1350 ± 440	0.368	< 0.001
Robust Mean Absolute Deviation	120 ± 52	103 ± 49	0.910	< 0.001
Uniformity	0.04 ± 0.01	0.05 ± 0.02	0.727	< 0.001
Root Mean Squared	613 ± 99	605 ± 103	0.961	< 0.001
Skewness	1.3 ± 1.0	1.3 ± 0.9	0.886	0.026
Total Energy	279,565,922 ± 477,324,746	269,270,070 ± 447,693,809	0.971	< 0.001
Variance	68,327 ± 38,406	51,160 ± 33,340	0.826	< 0.001
*Comparisons in the pre-IA subtype*				
10Percentile	−808 ± 81	−774 ± 85	0.894	< 0.001
90Percentile	−275 ± 232	−322 ± 219	0.908	< 0.001
Energy	8,206,450,953 ± 16,983,904,544	204,962,784 ± 450,423,006	−0.373	< 0.001
Entropy	4.9 ± 0.4	4.6 ± 0.5	0.803	< 0.001
Interquartile Range	256 ± 121	219 ± 111	0.863	< 0.001
Kurtosis	7.6 ± 5.1	7.0 ± 5.3	0.911	0.087
Maximum	718 ± 336	271 ± 335	0.446	< 0.001
Mean	−565 ± 122	−572 ± 121	0.983	0.004
Mean Absolute Deviation	172 ± 63	147 ± 60	0.831	< 0.001
Median	−604 ± 128	−607 ± 123	0.992	0.747
Minimum	−1021 ± 13	−985 ± 109	−0.214	< 0.001
Range	1739 ± 340	1257 ± 404	0.522	< 0.001
Robust Mean Absolute Deviation	110 ± 50	94 ± 46	0.858	< 0.001
Uniformity	0.04 ± 0.01	0.06 ± 0.02	0.810	< 0.001
Root Mean Squared	621 ± 96	614 ± 99	0.973	< 0.001
Skewness	1.4 ± 1.0	1.3 ± 0.9	0.941	0.223
Total Energy	202,069,467 ± 456,274,241	204,962,784 ± 450,423,007	0.879	< 0.001
Variance	60,456 ± 36,520	44,561 ± 31,243	0.758	< 0.001
*Comparisons in the IA subtype*				
10Percentile	−817 ± 112	−775 ± 112	0.796	< 0.001
90Percentile	−76 ± 220	−137 ± 215	0.936	< 0.001
Energy	20,646,241,681 ± 17,452,700,179	471,378,685 ± 376,597,456	0.732	< 0.001
Entropy	5.3 ± 0.4	5.1 ± 0.5	0.695	< 0.001
Interquartile Range	358 ± 131	304 ± 115	0.912	< 0.001
Kurtosis	5.1 ± 3.4	5.0 ± 2.8	0.850	< 0.001
Maximum	997 ± 499	547 ± 374	0.202	< 0.001
Mean	−493 ± 149	−500 ± 148	0.968	0.010
Mean Absolute Deviation	231 ± 58	200 ± 57	0.873	< 0.001
Median	−546 ± 180	−547 ± 173	0.966	0.729
Minimum	−1024 ± 0	−1097 ± 97	0.017	< 0.001
Range	2021 ± 499	1644 ± 419	0.223	< 0.001
Robust Mean Absolute Deviation	155 ± 52	132 ± 46	0.913	< 0.001
Uniformity	0.03 ± 0.01	0.04 ± 0.02	0.679	< 0.001
Root Mean Squared	589 ± 106	576 ± 112	0.956	< 0.001
Skewness	1.3 ± 0.9	1.0 ± 0.8	0.870	0.004
Total Energy	523,126,209 ± 464,115,141	471,378,651 ± 376,597,456	0.994	0.002
Variance	93,063 ± 33,657	71,899 ± 31,426	0.817	< 0.001

HR, high resolution. UHR, ultrahigh resolution

There were obvious discrepancies (all *ICC* < 0.60) between UHR and standard HR images in “Maximum,” “Minimum” and “Range.” “Energy” showed moderate consistency for the IA subtype (*ICC* = 0.732), but exhibited poor agreement in the pre-IA lesions (*ICC* = −0.373).

## Discussion

In this study, we compared the differences of subsolid nodule characteristics between low-dose UHR and standard HR images. The UHR images with 1024-matrix and 0.2-mm thickness prominently outperform standard HR images with 512-matrix and 1.0-mm thickness in terms of revealing quantitative and qualitative CT characteristics associated with invasive growth patterns of LUAD. Moreover, the ultrahigh-resolution scans on PCCT halved radiation exposure while maintaining superior image quality compared with conventional EID-CT.

Although previous studies using EID-CT indicated the utility of 1024-matrix UHR images in characterizing pulmonary nodule morphology [20, 21], these protocols required an additional targeted reconstruction or targeted scan of the nodule and brought high radiation doses (2.5–4.1 mSv). PCCT enables direct UHR acquisition of the entire lungs with 1024 matrix and 0.2-mm thickness at half the radiation dose (1.1–1.5 mSv), owing to the high pitch of 3.2, advantages of zero electronic noise and direct conversion of absorbed X-rays to electronic signals, unlike conventional EID-CT. In addition, Yanagawa, et al. [22] further employed EID-CT to generate whole-lung UHR images with 2048 matrix and 0.25-mm thickness for improved invasive lesions identification; however, this technique delivered an obviously higher radiation dose of (8.2 ± 1.8) mSv, and no comparison with standard HR was conducted.

In prior studies, heterogeneous attenuation, air bronchogram, and pleural indentation were found to be predictors of IA on UHR images [21, 22]. These imaging biomarkers were associated with the presence of pathologically invasive components of adenocarcinoma [2, 23, 24]. In this study, compared with standard HR, UHR images demonstrated an increased detection rate of heterogeneous attenuation, lobulation, bubble-like sign, pleural indentation, vascular sign and air bronchogram, attributable to its enhanced spatial resolution and minimized partial volume effect. Additionally, prior studies [18, 19, 25–27] also demonstrated the UHR protocol on PCCT allows subtle pulmonary structures to be clearly visible, for instance, higher-order bronchi, and is superior to fine reticulation and micronodules, among others, compared with conventional EID-CT. However, those studies were underscored on visualization of normal pulmonary structures and interstitial lung disease.

Our study further investigated the impact of PCCT on the observation of LUAD subtypes. In the subtype of pre-IA lesions, more lobulation, heterogeneous attenuation, bubble-like sign, air bronchogram and vascular sign were observed on UHR images compared to standard HR images. These morphological characteristics may lead to potential overestimation of invasiveness. Thus, a comprehensive assessment integrating quantitative measurements such as nodular diameter and solid-component diameter is important for accurately assessing invasiveness in subcentimeter nodules. As for the subtype of IA, UHR images demonstrated a higher prevalence of bubble-like sign, air bronchogram and pleural indentation than standard HR images, which could further enhance diagnostic confidence in malignancy. Additionally, CT findings including the absence of air bronchogram or bubble-like sign and the presence of pleural indentation were associated with an increased risk of metastasis in IA lesions [28–30].

Furthermore, previous preclinical studies using phantom models have demonstrated UHR imaging on PCCT provides more accurate nodular volume quantification compared with standard HR on EID-CT [31, 32]. In this study, measurements of both nodular diameter and solid-component diameter were statistically larger than those from standard HR images, possibly owing to the superior spatial resolution of UHR in delineating nodular margins. Notably, the ultrathin slice thickness of 0.2 mm from UHR images enabled identification of solid microfoci. In our cohort, 11 initially classified as pGGNs on standard HR images were recategorized as mGGNs on UHR images, with pathologically confirmed three IA, six MIA and two AIS. Conversely, three mGGNs observed on standard HR images were reclassified as pGGNs on UHR images including two AIS and one MIA, as the previously suspected solid-component microfoci on standard HR images corresponded to microvessels visualized through UHR images.

Histogram features provide objective quantitative parameters for evaluating tumor heterogeneity and attenuation characteristics. Previous studies demonstrated that histogram features, for instance “Variance,” “Skewness” were useful for identifying invasive lesions [33, 34]. In this study, UHR images provided a magnified tumor heterogeneity with larger signal intensity, energy and variability compared to standard HR images, attributable to the exponential increased pixel density from UHR acquisitions. Notably, significant discrepancies between UHR and HR images occurred in the “Maximum,” “Minimum,” “Range,” and “Energy” parameters within the pre-IA lesions. This phenomenon likely reflects UHR’s superior capacity to reveal air-containing areas with minimal attenuation and solid components with maximum attenuation compared to standard HR images. Consequently, UHR images demonstrated expanded attenuation ranges (“Maximum” and “Range”), and greater attenuation minima reduction (“Minimum”) in subsolid LUAD. Furthermore, the increased “Energy” in the pre-IA lesions on UHR images may be correlated with enhanced detection of heterogeneous attenuation and microsolid components below the spatial resolution threshold of conventional HR images.

This study had several limitations. First, this is a single-center and small-sample study; therefore, more cases are necessary in further studies to avoid bias. Second, the diagnostic accuracy of UHR for pathological LUAD subtypes needs further investigation in subsequent studies. Third, no comparison of visual and quantitative assessment was conducted between standard HR from EID-CT and UHR from PCCT, with the aim of avoiding repetitive scanning, although the image quality of standard HR images from PCCT outperformed that from EID-CT. Lastly, concerning the comparison between EID-CT and PCCT images, the scanning protocols were not unified due to the retrospective nature of our study, although PCCT yielded better image quality with a lower radiation dose.

In conclusion, the UHR images on PCCT significantly outperform standard HR images in terms of revealing the underlying CT characteristics of invasive LUAD and provide a magnified perspective of tumor heterogeneity, with advantages of halved radiation dose and better image quality than conventional EID-CT.

## Supplementary Information

Below is the link to the electronic supplementary material.

Supplementary file1 (DOCX 18 KB)
