# Supplementary material for: Low-dose ultrahigh-resolution PCCT enhances subsolid nodule characterization
Source: Radiol Med. 2025 Jul 29;130(8):1207–20. doi: 10.1007/s11547-025-02057-0 (PMC12367857; doi:10.1007/s11547-025-02057-0)
Supplement: Supplementary file 1 — Supplementary file1 (DOCX 18 KB) [file 11547_2025_2057_MOESM1_ESM.docx]

**Supplements**

**Table S1. Scanning protocols**

|  | Manufacture | Tube voltage / kVp | Tube current / mAs | FOV / cm | Matrix | Kernel | Slice-thickness / mm |
| --- | --- | --- | --- | --- | --- | --- | --- |
| PCCT | Siemens Healthineers | 120 | 171～380 | 31～42 | 1024 x 1024  512 x 512 | Bl60u\3 | 0.2  1.0 |
| Revolution | GE Medical System | 80 | 180～306 | 35～39 | 512 x 512 | Standard | 1.0 |
| Aquillion one | TOSHIBA | 100 | 176～261 | 30～41 | 512 x 512 | FC52 | 1.0 |
| uCT | United Imaging Healthcare | 120 | 183～330 | 30～40 | 512 x 512 | B_SHARP_C | 1.0 |

Note. PCCT, photon-counting CT. FOV: field of view.

Table S2. The inter-observer agreement of nodular characteristics on UHR and standard HR images.

|  | UHR images | | | Standard HR images | | |
| --- | --- | --- | --- | --- | --- | --- |
|  | Reader #1, % (n) | Reader #2, % (n) | Consistency | Reader #1, % (n) | Reader #2, % (n) | Consistency |
| Radiographic type, pGGN | 46.8 (95) | 44.8 (91) | 0.921 | 50.7 (103) | 51.2 (104) | 0.892 |
| Heterogeneous attenuation | 72.4 (147) | 76.8 (156) | 0.857 | 65.5 (133) | 61.1 (124) | 0.841 |
| Lobulation | 82.8 (168) | 82.3 (167) | 0.881 | 73.4 (149) | 68.0 (138) | 0.822 |
| Spiculation | 2.5 (5) | 2.5 (5) | 1.000 | 2.5 (5) | 2.5 (5) | 1.000 |
| Bubble-like sign | 25.1 (51) | 29.1 (59) | 0.900 | 8.9 (18) | 14.3 (29) | 0.735 |
| Air bronchogram | 37.9 (77) | 36.9 (75) | 0.916 | 20.2 (41) | 17.2 (35) | 0.741 |
| Pleural indentation | 21.2 (43) | 22.2 (45) | 0.913 | 16.3 (33) | 18.2 (37) | 0.896 |
| Vascular sign | 24.6 (50) | 34.0 (69) | 0.727 | 17.7 (36) | 25.6 (52) | 0.623 |
| Nodular.diameter /mm  IQR  Mean ± SD | 10.1 (8.3, 10.4)  11.7 ± 4.8 | 10.6 (8.4, 14.3)  12.0 ± 4.8 | 0.974 | 10.0 (8.1, 13.4)  11.3 ± 4.8 | 10.4 (8.1, 13.9)  11.6 ± 4.8 | 0.966 |
| Solid-component diameter /mm  IQR  Mean ± SD | 2.1 (0, 6.0)  3.6 ± 4.7 | 2.0 (0, 5.2)  3.5 ± 4.6 | 0.969 | 0 (0, 5.6)  3.4 ± 4.7 | 0 (0, 5.1)  3.2 ± 4.5 | 0.952 |
| CT value /HU  IQR  Mean ± SD | -470 (-606, -318)  -450 ± 198 | -466 (-597, -273)  -432 ± 205 | 0.941 | -484 (-604, -338)  -466 ± 185 | -479 (-601, -338)  -460 ± 184 | 0.947 |
| SD / HU  IQR  Mean ± SD | 151 (99, 249)  176 ± 91 | 142 (100, 229)  171 ± 90 | 0.858 | 146 (89, 221)  163 ± 89 | 143 (92, 216)  163 ± 89 | 0.875 |

Note. pGGN, pure ground-glass nodule. SD, standard deviation. HR, high resolution. UHR, ultrahigh resolution. IQR, interquartile range. HU, Hounsfield unit.
